# The influence of COVID-19 barrier measures on the positivity rate of typhoidal salmonellosis and amoebiasis in the Buea Health District, South West Region of Cameroon

**DOI:** 10.1371/journal.pgph.0001854

**Published:** 2023-04-26

**Authors:** Afegenui Blaise Sunday, Raymond Babila Nyasa, Martin Mokake

**Affiliations:** 1 Department of Public Health and Hygiene, University of Buea, Buea, Cameroon; 2 Department of Microbiology and Parasitology, University of Buea, Buea, Cameroon; 3 Department of Surgery and Obstetrics and Gynaecology, University of Buea, Buea, Cameroon; Christian Medical College Vellore, INDIA

## Abstract

Typhoidal salmonellosis and amoebiasis are prevalent in the Buea Health District (BHD) and it is evident that hand hygiene can reduce the transmissibility of these diseases. The barrier measures enforced by the government, in the wake of the corona virus disease 2019 (COVID-19) pandemic has led to behavioural changes which may have had an influence on the positivity rate of these diseases. This study seeks to determine the influence of COVID-19 barrier measures and the implementation of COVID-19 vaccination on the positivity rate of typhoidal salmonellosis and amoebiasis in the BHD. A retrospective study, with purposive and random sampling methods were used to select health facilities in BHD, whose laboratory records were reviewed using a data extraction form to obtain health information of patients who tested for typhoidal salmonella and/or *Entamoeba histolytica* from June 1, 2018 to December 31, 2021. Chi-square test was used to compare the positivity rate in the pre-and-COVID-19 and in the pre-and-vaccination era. The positivity rate of typhoidal salmonellosis and amoebiasis dropped from 57.8% and 2.7% in the pre-COVID-19 era to 34% and 1.8% in the COVID-19 era respectively, which were significant (χ^2^ = 945.8; P<0.001 and χ^2^ = 11.8; P = 0.001 respectively). Within the COVID-19 era, the positivity rate of salmonellosis and amoebiasis decreased from 39% and 2.6% before the implementation of COVID-19 vaccination to 27.7% and 0.8% respectively in the COVID-19 vaccination era and these differences were significant (χ^2^ = 149.1; P<0.001 and χ^2^ = 33.8; P<0.001 respectively). However, the positivity rate of salmonellosis between the rainy (43.5%) and the dry (42.8%) seasons and amoebiasis between the rainy (2.2%) and the dry (2%) seasons were not significantly different (χ^2^ = 0.6; P = 0.429 and χ^2^ = 0.54; P = 0.463 respectively). A significant decline in the positivity rate of typhoidal salmonellosis and amoebiasis was observed after the implementation of COVID-19 barrier measures and vaccination.

## Introduction

The World Health Organization (WHO) has estimated that annually there are 16.6 million cases of typhoid fever with about 600000 deaths worldwide [1]. Also in tropical and temperate regions about 50 million people are infected with amoebiasis, and annually 100,000 deaths occur worldwide [2, 3]. A study carried out in Buea indicated that there was a high prevalence of salmonellosis and amoebiasis of 39.7% and 24.4%, respectively [4, 5]. Typhoidal salmonellosis and amoebiasis are commonly transmitted through the faeco-oral route, which is enhanced by poor hand washing habits and personal hygiene and most inhabitants of the BHD face difficulties getting clean water for hygiene and sanitation especially in the dry season. Studies carried out in the Buea Health District (BHD), suggested that the transmission of intestinal protozoans are not only active, but on a rise [5].

Coronavirus disease 2019 (COVID-19) is an emerging respiratory disease that was first reported to the World Health Organization (WHO) as a cluster of pneumonia of unknown origin from Wuhan, China, in December 2019, and the first case of COVID-19 was diagnosed in Cameroon on March 6, 2020 [6]. Good sanitation and hygiene were highlighted by WHO as major ways to curb the spread of COVID-19 [7–9]. In this light, the government of Cameroon on March 17, 2020 put in place measures to curb the spread of COVID-19, which went operational as from March 18, 2020, especially regular hand washing with soap, avoid close contact such as shaking hands or hugging and covering the mouth when sneezing [10]. There was a wave of intensive sensitization and galvanization so that the measures put in place by the governments especially hand hygiene and no handshakes were closely followed especially at the beginning of the pandemic as was observed generally in the city of Buea. It was suggested that these multi‑modal infection control strategies which were designed to contain the COVID‑19 pandemic may have an unintended positive effect on other infections such as salmonellosis and amoebiasis. Vaccination was later implemented as a protective measure on April 12, 2021 and BHD had a number of vaccination centres [6]. The psychological consolation of the Buea population, due to the availability of a vaccine against the dreaded COVID-19 may have caused a relaxation in the implementation of barrier measures, which happens to be preventive measures of salmonellosis and amoebiasis transmission. This study investigated the positivity rate of salmonellosis and amoebiasis in the pre-covid-19 and COVID-19 era in BHD, and further investigated the influence of COVID-19 vaccination on the positivity rate of salmonellosis and amoebiasis in the COVID-19 era.

## Materials and methods

### Study area and setting

Buea is the administrative headquarters of the South West Region of Cameroon, one of the regions plagued by diarrhoeal diseases especially salmonellosis and amoebic dysentery. Buea is a town located on the Eastern slopes of Mount Cameroon and lies between latitudes 40 12°N and longitudes 90 12°E with a total surface area of 870 km^2^. The total population was estimated to be over 200,000 inhabitants in 2020 [11]. With reference to occupation, the population is mainly composed of students, civil servants and traders. Buea is one of the fastest growing towns in Cameroon today with a mixed cosmopolitan setting. The Buea Health District has 36 functional Health facilities. This health district faces a serious problem of water supply to meet the needs of the growing population making good hygiene and sanitation difficult to achieve. The BHD is characterised by many churches, schools, bars, restaurants, nightclubs and other avenues that bring together large populations. These areas in the wake of the COVID-19 pandemic were given the ultimatum by the prime minister to have water and soap at the entrance for washing of hands or hand sanitizers for their clients, maintain social distancing of at least one metre and no handshakes. The BHD set up two centres for COVID-19 vaccination and also had other mobile vaccination strategies implemented.

### Study design

This study was a hospital-based retrospective study, which involved a comprehensive review of patients’ records who were tested for Salmonella species and *Entamoeba histolytica* from June 1, 2018 to December 31, 2021.The aim of this study was to compare the positivity rate of typhoidal salmonellosis and amoebiasis in the pre-COVID-19 era from June 1, 2018 to March 5, 2020 (when the first case of COVID-19 was reported in Cameroon) to that of the COVID-19 era, from March 6, 2020 to December 31, 2021. Within the COVID-19 era, comparison was made on the positivity rate of typhoidal salmonellosis and amoebiasis, before COVID-19 vaccination and after COVID-19 vaccination, which started in Buea on April 12, 2021.

Simple random and purposive sampling methods were used to select health facilities from different levels of health care in the BHD. Firstly, the Regional Hospital Buea (RHB) was selected purposively since it is the only secondary level referral health facility in the BHD and the South West Region at large. Secondly, the Muea Medicalized Health Centre (MMHC) was also purposively selected, which is the only public medicalized health centre of the district. The Mount Mary Hospital (MMH) was randomly selected from the two confessional hospitals of the district (Seventh Day Adventist hospital and Mount Mary Hospital). Furthermore, a simple random sampling method was used on all integrated health centres and the Buea Town Integrated Health Centre (BTIHC) was selected. Lastly, the Solidarity Hospital was selected from all the private health facilities by simple random sampling. After going through the records of Solidarity Hospital, it was found that they were incomplete and another simple random sampling of the private health facilities was done and the Access Care health centre (ACHC) was selected. So, in all, five (5) functional health facilities were selected from the 36 that exist in the BHD.

### Ethics statement

Ethical clearance for this study was obtained from the Faculty of Health Sciences- Institutional Review Board of the University of Buea, reference number 2022/1615-01/UB/SG/IRB/FHS and administrative authorization was obtained from the Regional Delegation for the South West Region, reference number R11/MINSANTE/SWR/RDPH/PS/300/305. Administrative authorization was also obtained from the District Health Service Buea, reference number F_1_Vol_2_/L/MINSANTE/RDPHSW/DHS/Buea/106a. Written formal permissions were obtained from heads of the ethics review committees of these health facilities in which they waived obtaining informed consent from patients to access data as has been done in other research works [12–14]. The data was collected anonymously and confidentiality was maintained by the non-identification of patients’ information.

### Data collection

An electronic data extraction form was designed on EPI INFO version 7.2 and used to extract information from laboratory registers on age, sex, date and results of all patients who submitted samples to the laboratory for diagnosis of typhoidal salmonellosis and amoebiasis between June 1, 2018 to December 31, 2021. In BHD, typhoidal salmonellosis is predominantly diagnosed by serology (widal test and rapid diagnostic test), stool culture, and blood culture, while amoebiasis is diagnosed by stool microscopy. Data extraction was conducted between December 2021 to June 2022 and a total sample size of 17274 for typhoidal salmonellosis and 11972 for amoebiasis was obtained.

### Statistical analysis

Data obtained using EPI INFO version 7.2 was exported and analysed on Microsoft-Excel 2016 (S1 and S2 Data) and SPSS version 25. Demographic data was presented using bar charts. The chi-square statistical test was used to compare the positivity rate of typhoidal salmonellosis and amoebiasis in the pre-COVID-19 and COVID-19 era and within the COVID-19 era it was also used to compare the positivity rate of typhoidal salmonellosis and amoebiasis of the period before and during the implementation of COVID-19 vaccination. The chi-square test was also used to determine the association between positivity rate of these diseases and seasonality (the dry and rainy seasons) which is the period between March-October for the rainy and November-February for the dry seasons and also for the different periods in the different health facilities. P-values of ≤0.05 was said to be statistically significant.

## Results

### File selection and characteristics

**Fig 1:**
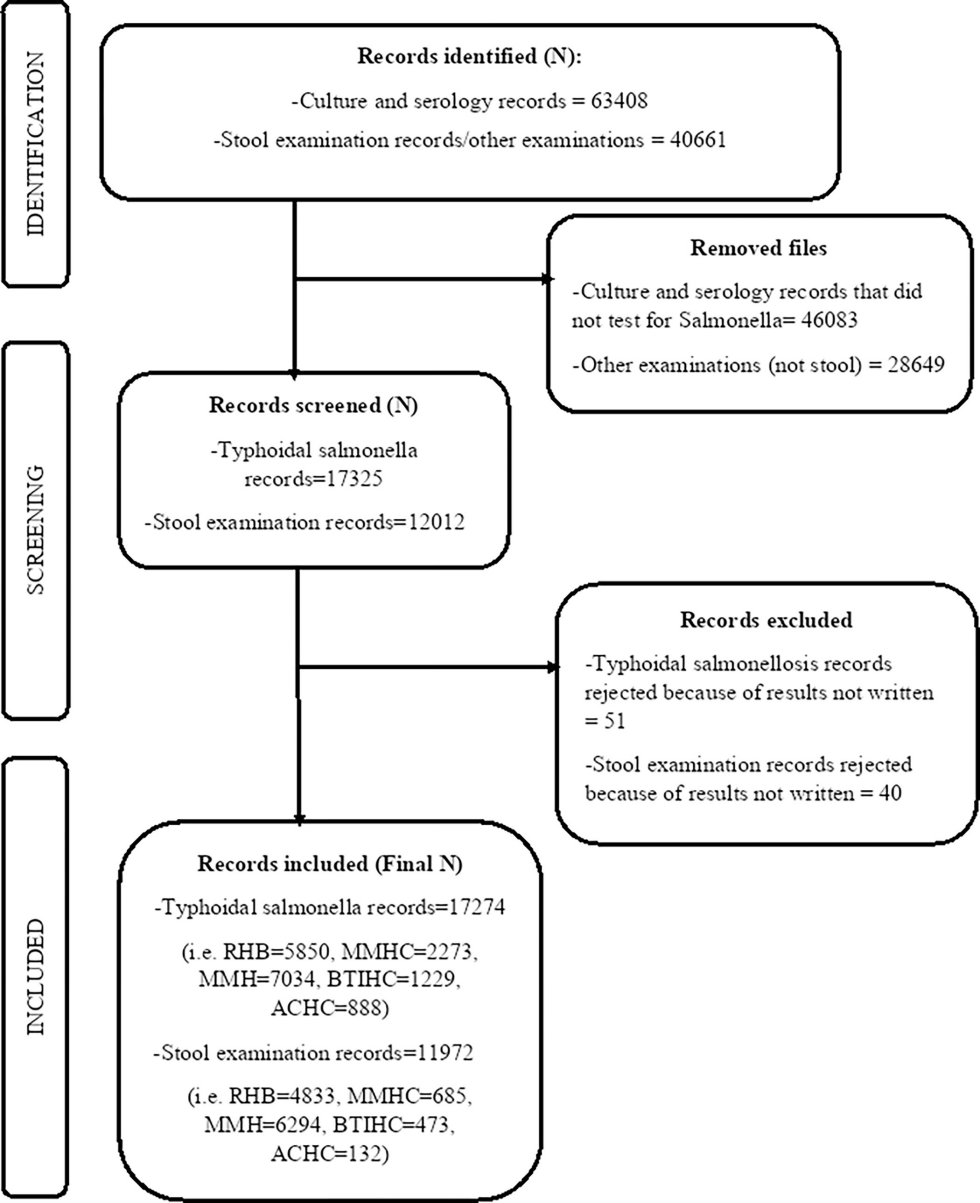
Flowchart of sample size determination.

The culture and serology registers were consulted for typhoidal salmonellosis in the selected health facilities and 63408 files were found as illustrated in Fig 1 i.e. 27865 (43.9%) from the RHB, 7611 (12%) from the MMHC, 20823 (32.8%) from the MMH, 4650 (7.3%) from the BTIHC and 2459 (3.9%) from the ACHC. Amongst these files, 17325 contained data on typhoidal salmonellosis but 51 (0.3%) were rejected because the results of these tests were not recorded leaving a sample size of 17274 i.e. 5850 (33.9%) in the RHB, 2273 (13.2%) in the MMHC, 7034 (40.7%) in the MMH, 1229 (7.1%) in the BTIHC and 888 (5.1%) in the ACHC amongst which 7468 were positive. Likewise, the stool examination register was consulted for amoebiasis in the selected health facilities and 40661 files were found (Fig 1) i.e. 11457 (28.2%) from the RHB, 9015 (22.2%) from the MMHC, 12889 (31.7%) from the MMH, 4692 (11.5%) from the BTIHC and 2608 (6.4%) from the ACHC. From this total, 12012 files contained data on amoebiasis but 40 (0.3%) were rejected because the results of these tests were not recorded leaving a sample size of 11972 i.e. 4388 (36.7%) in the RHB, 685 (5.7%) in the MMHC, 6294 (52.6%) in the MMH, 473 (4%) in the BTIHC and 132 (1.1%) in the ACHC amongst which 260 were positive in the BHD.

### Distribution of typhoidal salmonellosis and amoebiasis cases according to age groups in the BHD

**Fig 2:**
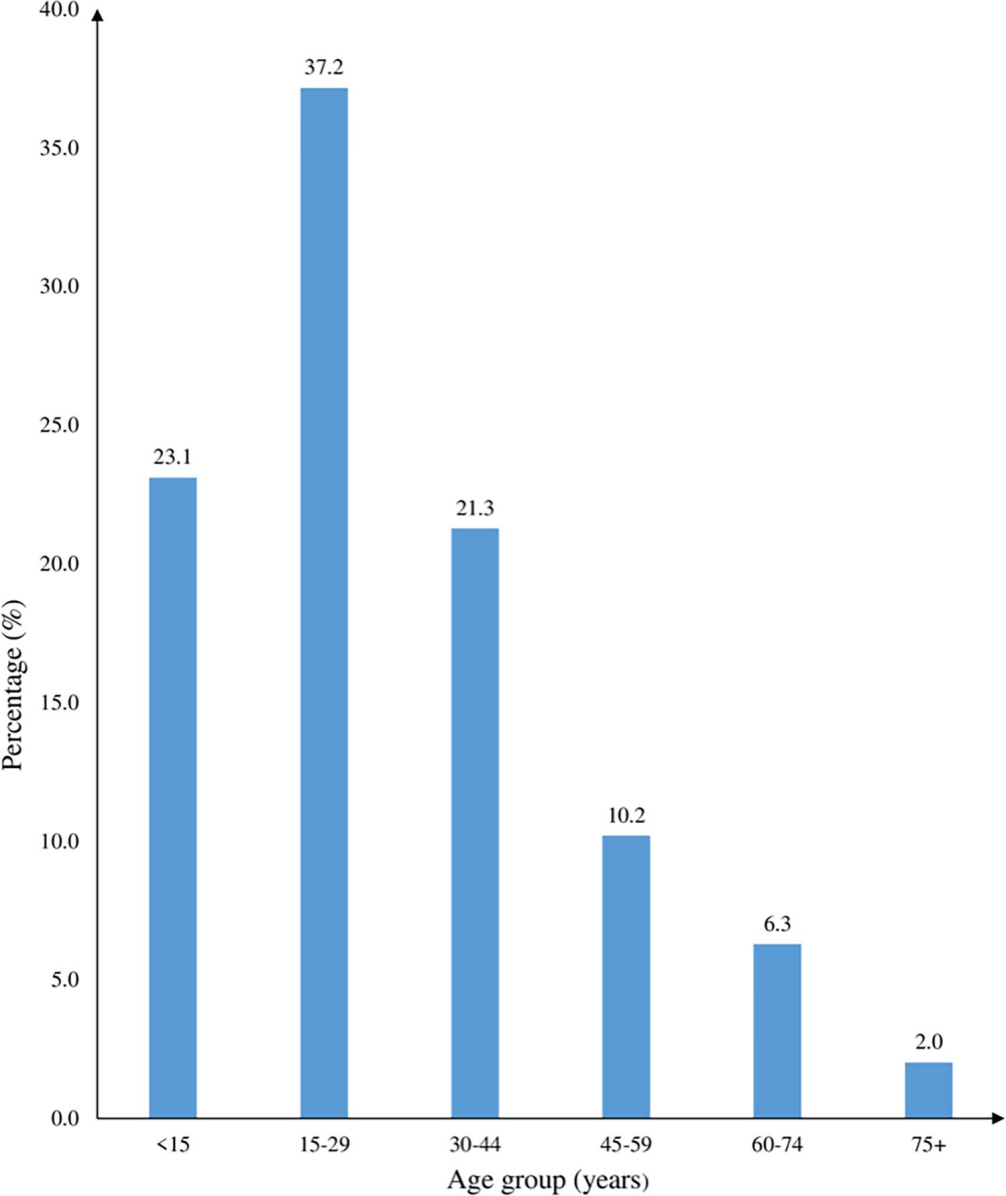
Distribution of typhoidal salmonellosis cases by age group in the BHD.

The majority of the cases of salmonellosis as shown in Fig 2 were within the age group of 15–29 years (37.2%) followed by those less than 15 years (23.1%) and those of the age group 30–44 years (21.3%).

**Fig 3:**
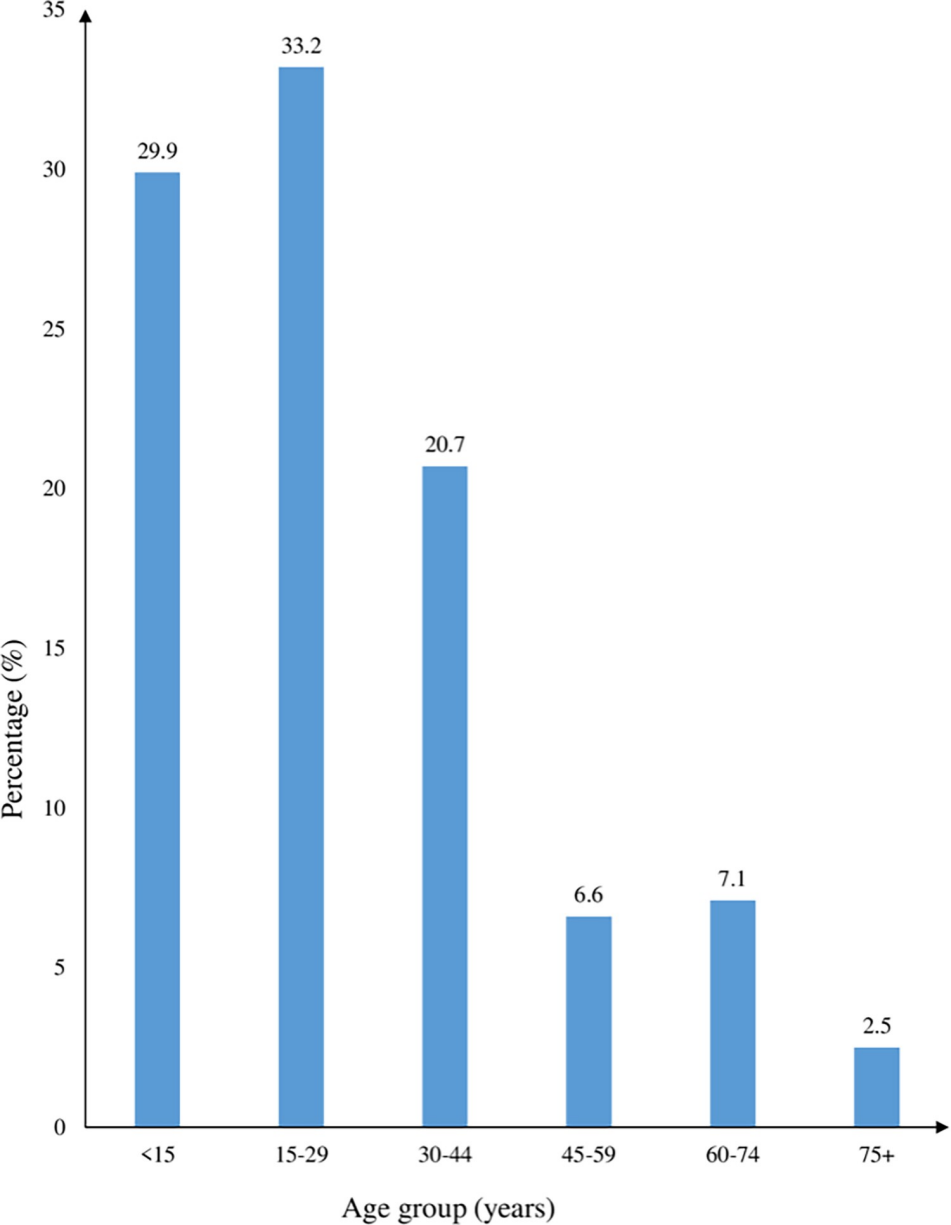
Distribution of amoebiasis cases by age group in the BHD.

The majority of amoebiasis cases as shown in Fig 3 were within the age group 15–29 years (29.9%) followed by the age group less than 15 years (29.9%) and then 30-44years (20.7%).

### Positivity rate of typhoidal salmonellosis before and during the COVID-19 era in the BHD

Generally, the total number of typhoidal salmonellosis cases in the pre-COVID-19 era were 6683 amongst which 3865 were positive leaving a positivity rate of 57.8%. Also, the COVID-19 era recorded a total of 10591 typhoidal salmonellosis cases amongst which 3603 were positive resulting to a positivity rate of 34%. This decrease from 57.8% in the pre-COVID-19 era to 34% in the COVID-19 era was statistically significant (χ^2^ = 945.8 and P<0.001) as shown in Table 1. Specifically, the positivity rate of typhoidal salmonellosis decreased from the pre-COVID-19 era (30.4%) to the COVID-19 era (19.4%) in the RHB which was statistically significant (χ^2^ = 90.6 and P<0.001). Also, the positivity rate of salmonellosis decreased from the pre-COVID-19 era (74.2%) to the COVID-19 era (26.2%) in the Mount Mary Hospital and this difference was statistically significant (χ^2^ = 536.7 and P<0.001). However, the ACHC which had a positivity rate of 86.7% in the pre-COVID-19 era and 85.9% in the COVID-19 era showed no statistically significant difference (χ^2^ = 0.108, p = 0.743). Meanwhile for the MMHC, the positivity rate in the COVID-19 era (68.4%) was more than the pre-COVID-19 (62.7%) and this difference was statistically significant (χ^2^ = 7.8 and P = 0.005). In BTIHC the positivity rate also increased from 58.5% in the pre-COVID-19 era to 63.9% in the COVID-19 era, but the difference was not statistically significant (χ^2^ = 3.6, p = 0.059).

**Table 1:** Positivity rate of typhoidal salmonellosis per health facility before and during the COVID-19 era in the BHD.

Health Facility	Period	N° tested (%)	N° Positive (%)	N° Negative (%)	Positivity rate (%)	χ^2^	P-value
RHB	Pre-COVID-19	2143 (12.4)	652 (3.8)	1491 (8.6)	30.4	90.6	<0.001
	COVID-19	3707 (21.5)	721 (4.2)	2986 (17.3)	19.4		
	**Total**	**5850 (33.9)**	**1373 (7.9)**	**4477 (25.9)**	**23.5**		
MMHC	Pre-COVID-19	1043 (6.0)	654 (3.9)	389 (2.3)	62.7	7.8	0.005
	COVID-19	1230 (7.1)	841 (4.9)	389 (2.3)	68.4		
	**Total**	**2273 (13.2)**	**1495 (8.7)**	**778 (4.5)**	**65.8**		
MMH	Pre-COVID-19	2575 (14.9)	1911 (11.1)	664 (3.8)	74.2	536.7	<0.001
	COVID-19	4459 (25.8)	1167 (6.8)	3292 (19.1)	26.2		
	**Total**	**7034 (40.7)**	**3078 (17.8)**	**3956 (22.9)**	**43.8**		
BTIHC	Pre-COVID-19	547 (3.2)	320 (1.9)	227 (1.3)	58.5	3.6	0.059
	COVID-19	682 (3.9)	436 (2.5)	246 (1.4)	63.9		
	**Total**	**1229 (7.1)**	**756 (4.4)**	**473 (2.7)**	**61.5**		
ACHC	Pre-COVID-19	376 (2.2)	326 (1.9)	50 (0.3)	86.7	0.108	0.743
	COVID-19	512 (3.0)	440 (2.5)	72 (0.4)	85.9		
	**Total**	**888 (5.1)**	**766 (4.4)**	**122 (0.7)**	**86.4**		
**Total (BHD)**	Pre-COVID-19	6684 (38.7)	3863 (22.4)	2821 (16.3)	57.8	945.8	<0.001
	COVID-19	10590 (61.3)	3605 (20.9)	6985 (40.5)	34		
	**Total**	**17274 (100)**	**7468 (43.2)**	**9806 (56.8)**	**43.2**		

N° = Number, % = Percentage, χ^2^ = Chi-square, ACHC = Access Care Health Centre, BTIHC = Buea Town Integrated Heath Centre, MMH = Mount Mary Hospital, MMHC = Muea Medicalized Health Centre, RHB = Regional Hospital Buea.

### Positivity rate of amoebiasis before and during the COVID-19 in the BHD

Overall, the total number of amoebiasis cases in the pre-COVID-19 era were 4864 amongst which 133 were positive making a positivity rate of 2.7%. Also, in the COVID-19 era, 7108 cases were recorded and 127 were positive resulting to a positivity rate of 1.8%. This decrease in the positivity rate of amoebiasis from 2.7% in the pre-COVID-19 era to 1.8% in the COVID-19 era was statistically significant (χ^2^ = 11.8 and p = 0.001) Table 2. Specifically, in RHB, the positivity rate decreased from 6% in the pre-COVID-19 era to 3.3% in the COVID-19 era, which was statistically significant (χ^2^ = 18.4, p<0.001). On the contrary, it was observed in MMHC that the positivity rate of amoebiasis increased from 0.3% in the pre-COVID-19 era to 3.9% in the COVID-19 era, and this difference was statistically significant (χ^2^ = 7.8, p = 0.005). There was a general decrease in the positivity rate of amoebiasis from the pre-COVID-19 era to the COVID-19 era for MMH, BTIHC and ACHC, but the differences were not statistically significant.

**Table 2:** Positivity rate of amoebiasis per health facility before and during the COVID-19 era in the BHD.

Health Facility	Period	N° tested (%)	N° Positive (%)	N° Negative (%)	Positivity rate (%)	χ^2^	P-value
RHB	Pre-COVID-19	1660 (13.9)	100 (0.8)	1560 (13.0)	6	18.4	<0.001
	COVID-19	2728 (22.8)	89 (0.7)	2639 (22.0)	3.3		
	**Total**	**4388 (36.7)**	**189 (1.6)**	**4199 (35.1)**	**4.3**		
MMHC	Pre-COVID-19	298 (2.5)	1 (0.0)	297 (2.5)	0.3	7.8	0.005
	COVID-19	387 (3.2)	15 (0.1)	372 (3.1)	3.9		
	**Total**	**685 (5.7)**	**16 (0.1)**	**669 (5.6)**	**2.3**		
MMH	Pre-COVID-19	2616 (21.9)	7 (0.1)	2609 (21.8)	0.3	1.4	0.238
	COVID-19	3678 (30.7)	4 (0.0)	3674 (30.7)	0.1		
	**Total**	**6294 (52.6)**	**11 (0.1)**	**6283 (52.5)**	**0.2**		
BTIHC	Pre-COVID-19	211 (1.8)	3 (0.0)	208 (1.7)	1.4	0.5	0.47
	COVID-19	262 (2.2)	1 (0.0)	261 (2.2)	0.4		
	**Total**	**473 (4.0)**	**4 (0.0)**	**469 (3.9)**	**0.8**		
ACHC	Pre-COVID-19	79 (0.7)	22 (0.2)	57 (0.5)	27.8	0.3	0.578
	COVID-19	53 (0.4)	18 (0.2)	35 (0.3)	34		
	**Total**	**132 (1.1)**	**40 (0.3)**	**92 (0.8)**	**30.3**		
**Total (BHD)**	Pre-COVID-19	4864 (40.6)	133 (1.1)	4731 (39.5)	2.7	11.8	0.001
	COVID-19	7108 (59.4)	127 (1.1)	6981 (58.3)	1.8		
	**Total**	**11972(100)**	**260 (2.2)**	**11712(97.8)**	**2.2**		

N° = Number, % = Percentage, χ^2^ = Chi-square, ACHC = Access Care Health Centre, BTIHC = Buea Town Integrated Heath Centre, MMH = Mount Mary Hospital, MMHC = Muea Medicalized Health Centre, RHB = Regional Hospital Buea.

### Positivity rate of typhoidal salmonellosis before and after the introduction of COVID-19 vaccine, in the COVID-19 era, within the BHD

The positivity rate of typhoidal salmonellosis decreased from the period before COVID-19 vaccination (39.1%) to 27.7% during the COVID-19 vaccination period, which was statistically significant (χ^2^ = 149.1; P<0.001), Table 3. Likewise, the positivity rate of amoebiasis decreased from 2.6% in the period before COVID-19 vaccination to 0.8% during COVID-19 vaccination period and this difference was statistically significant (χ^2^ = 33.8, p<0.001).

**Table 3:** Positivity rate of typhoidal salmonellosis and amoebiasis in the BHD before and during the vaccination periods of the COVID-19 era.

Disease	COVID-19 Period	N° tested (%)	N° positive (%)	N° negative (%)	Positivity rate (%)	χ^2^	P-value
Salmonellosis	Before vaccination	5899 (55.7)	2305 (21.8)	3594 (33.9)	39.1	149.1	<0.001
	During Vaccination	4691 (44.3)	1300 (12.3)	3391 (32.0)	27.7		
	**Total**	**10590 (100)**	**3605 (34)**	**6985 (66)**	**34**		
Amoebiasis	Before vaccination	3872 (54.5)	102 (1.4)	3770 (53.0)	2.6	33.8	<0.001
	During Vaccination	3236 (45.5)	25 (0.4)	3211 (45.2)	0.8		
	**Total**	**7108 (100)**	**127 (1.8)**	**6981 (98.2)**	**1.8**		

N° = Number, % = Percentage, χ^2^ = Chi-square.

### Seasonal influence on the positivity rate of typhoidal salmonellosis within the BHD

The positivity rate of typhoidal salmonellosis between the rainy (43.5%) and the dry (42.8%) seasons in BHD, were not statistically significantly different (χ^2^ = 0.6; p = 0.429), Table 4. Specifically, there was no statistically significant difference between the rainy and the dry season in the pre-COVID-19 era (χ^2^ = 0.1, p = 0.773), likewise during the COVID-19 vaccination period (χ^2^ = 0.4; p = 0.552), but there was a significant difference between the rainy and the dry season in the period before vaccination within the COVID-19 era (χ^2^ = 46.4, p<0.001).

**Table 4:** Seasonal distribution of the positivity rate of typhoidal salmonellosis in the BHD.

Period	Season	N° tested (%)	N° positive (%)	N° negative (%)	Positivity rate (%)	χ^2^	P-value
Pre-COVID-19	Rainy	4082 (23.6)	2353 (13.6)	1729 (10.0)	57.6	0.1	0.773
	Dry	2602 (15.1)	1510 (8.7)	1092 (6.3)	58		
	**Total**	**6684 (38.7)**	**3863 (22.4)**	**2821 (16.3)**	**57.8**		
COVID-19 (BV)	Rainy	3990 (23.1)	1679 (9.7)	2311 (13.4)	42.1	46.4	<0.001
	Dry	1909 (11.1)	626 (3.6)	1283 (7.4)	32.8		
	**Total**	**5899 (34.1)**	**2305 (13.3)**	**3594 (20.8)**	**39.1**		
COVID-19 (DV)	Rainy	3276 (19.0)	899 (5.2)	2377 (13.8)	27.4	0.4	0.552
	Dry	1415 (8.2)	401 (2.3)	1014 (5.9)	28.3		
	**Total**	**4691 (27.2)**	**1300 (7.5)**	**3391 (19.6)**	**27.7**		
**Total**	Rainy	11348 (65.7)	4931 (28.5)	6417 (37.1)	43.5	0.6	**0.429**
	Dry	5926 (34.3)	2537 (14.7)	3389 (19.6)	42.8		
	**Total**	**17274 (100)**	**7468 (43.2)**	**9806 (56.8)**	**43.2**		

N° = Number, % = Percentage, χ^2^ = Chi-square, BV = Before vaccination, DV = During Vaccination.

#### Seasonal influence on the positivity rate of amoebiasis within the BHD

The positivity rate of amoebiasis between the rainy (2.2%) and the dry (2%) seasons in BHD, were not statistically significantly difference (χ^2^ = 0.54 and P = 0.463), Table 5. Specifically, there was no statistically significant difference between the rainy and the dry seasons in the pre-COVID-19 era (χ^2^ = 0.03, p = 0.871), and in the period during vaccination of the COVID-19 era (χ^2^ = 0.55, p = 0.458), but there was a significant difference in the period before vaccination of the COVID-19 era (χ^2^ = 4.3, p = 0.038) in the BHD.

**Table 5:** Seasonal distribution of the positivity rate of amoebiasis in the BHD.

Period	Season	N° tested (%)	N° positive (%)	N° negative (%)	Positivity rate (%)	χ^2^	P-value
Pre-COVID-19	Rainy	2977 (24.9)	80 (0.7)	2897 (23.2)	2.7	0.03	0.871
	Dry	1887 (15.8)	53 (0.4)	1834 (15.3)	2.8		
	**Total**	**4864 (40.6)**	**133 (1.1)**	**4731 (39.5)**	**2.7**		
COVID-19 (BV)	Rainy	2821 (23.6)	84 (0.7)	2737 (22.9)	2.9	4.3	0.038
	Dry	1051 (8.8)	18 (0.2)	1033 (8.6)	1.7		
	**Total**	**3872 (32.3)**	**102 (0.9)**	**3770 (31.5)**	**2.6**		
COVID-19 (DV)	Rainy	2448 (20.4)	21 (0.2)	2427 (20.3)	0.9	0.55	0.458
	Dry	788 (6.6)	4 (0.0)	784 (6.5)	0.5		
	**Total**	**3236 (27.0)**	**25 (0.2)**	**3211 (26.8)**	**0.8**		
**Total**	Rainy	8246 (68.9)	185(1.5)	8061 (67.3)	2.2	0.54	**0.463**
	Dry	3726 (31.1)	75 (0.6)	3651 (30.5)	2		
	**Total**	**11972 (100)**	**260 (2.2)**	**11712 (97.8)**	**2.2**		

N° = Number, % = Percentage, χ^2^ = Chi-square, BV = Before vaccination, DV = During Vaccination.

## Discussion

The positivity rate of typhoidal salmonellosis and amoebiasis significantly reduced in the COVID-19 era compared to the pre-COVID-19 era in the BHD. This significant reduction may be explained by the implementation of barrier measures in the BHD to curb the spread of COVID-19 amongst which included handwashing with soap, no handshakes and the use of hand sanitizers. This is in agreement with previous observation that washing hands with soap can reduce the risk of diarrhoeal diseases by 42–47% and interventions to promote handwashing might save a million lives [15]. This also confirms the finding that the multimodal infection control strategies designed to contain the COVID-19 pandemic had an unintended positive effect on other infections [16].

Within the COVID-19 era, the positivity rate of typhoidal salmonellosis and amoebiasis was significantly lower during the vaccination period of COVID-19 compared to the period before vaccination. This is contrary to the hypothesis that was suggested at the beginning of this study; that the introduction of vaccination against COVID-19 may cause the relaxation in the implementation of the barrier measures. However, the significant drop in typhoidal salmonellosis and amoebiasis during the vaccination period of COVID-19 may be explained by the low vaccine uptake of 17.1% [17] for the COVID-19 vaccines (vaccine hesitancy) which according to MacDonald is context- and vaccine-specific [18]. In terms of context, it was more as a result of anti-vaccine campaigns spread over social media which probably led the population of the BHD to prefer intensifying the barrier measures rather than seeking protection from the COVID-19 vaccines. To add, it is also in line with the findings of Caserotti *et al*. in 2021 that the more doubtful people are about vaccines in general, the less willing they were to get vaccinated, no matter the specific vaccine. It seems reasonable that the more people have doubts about vaccines in general, the less they are likely to be willing to undergo vaccinations of any kind [19].

There was no significant difference between the dry and the rainy season in the positivity rate of typhoidal salmonellosis and amoebiasis in the BHD. This confirms previous findings that in Cameroon and the Democratic Republic of Congo, a more constant occurrence of typhoidal salmonellosis has been described with no distinct seasonal cycle [20, 21]. This is contrary to what has been observed in Blantyre, Malawi where a distinct seasonal cycle of salmonellosis exists with peak in March-June following the rainy season [22, 23].

The strength of this work is that, it shows the need/importance of intensifying hand hygiene and no handshakes in the BHD where there is the high prevalence of typhoidal salmonellosis and amoebiasis and also solves the problem of paucity of data in this area. Despite these strengths, there were some limitations; firstly, blood and stool cultures would have been the ideal data collected, but because the RHB is the only health facility using these diagnostic methods, Widal and rapid diagnostic tests were also considered for this study, since they are equally scientifically accepted diagnostic methods for typhoidal salmonellosis especially in places where culture cannot be achieved. Secondly, other confounding factors such as source of drinking water and contamination of food by rodents and flies on the one hand and less people moving outside for work and less food consumption from public restaurants on the other hand may have had an impact on the results of the study. Nevertheless, the role of these barrier measures in the reduction of the positivity rate of typhoidal salmonellosis and amoebiasis cannot be underestimated.

In conclusion, by using the time points of the sequelae of events that occurred in the BHD with the emergence of COVID-19, we observed that the implementation of the COVID-19 barrier measures, amongst which include regular hand washing with soap and no hand shaking, may have led to a significant decrease in the positivity rates of typhoidal salmonellosis and amoebiasis. However, after vaccines were initiated, there was a decrease in the positivity rate of typhoidal salmonellosis and amoebiasis. Thus, the public should be sensitized on the secondary health benefits implicit in the implementation of COVID-19 barrier measures.

Further research should focus on the microbiological quality of the different water sources consumed within the BHD. Also restaurant owners should be periodically screened for enteric pathogens and their hygienic practices in food preparation should be evaluated, to determine its contribution to the spread of salmonellosis and amoebiasis in BHD.

## Supporting information

S1 Data Typhoidal salmonellosis data June 2018-Dec 2021. UniqueKey [Serial number], Healthfacilitylaboratory [Health facilities/laboratories (MMH = Mount Mary Hospital, RHB = Regional Hospital Buea, ACHC = Access Care Health Centre, MHC = Medicalized Health Centre, BTIHC = Buea Town Integrated Health Centre)], Age [Age in years], Sex [0 for male and 1 for female], Q4Year [Year test was carried out], Q5Month [Month test was carried out (0 = January, 1 = February, 2 = March, 3 = April, 4 = May, 5 = June, 6 = July, 7 = August, 8 = September, 9 = October, 10 = November, 11 = December)], Weekofthemonth [Week of the month test was carried out (0 = week 1, 1 = week 2, 2 = week 3, 3 = week 4, 4 = week 5, 5 = week 6)], Dayofthemonth [Date of the month test was carried out], Typeoftest [Kind of test carried out (0 = Widal, 1 = Blood culture, 2 = Stool culture, 3 = Others)], Salmonellastatus [Test result (0 = Positive and 1 = Negative)], Q10Ifpositivestrain [Specification of salmonella strain when test result was positive (0 = Typhi, 1 = Paratyphi, 2 = Both typhi and paratyphi)], ParatyphiA [Salmonella paratyphoid caused by paratyphi A], ParatyphiB [Salmonella paratyphoid caused by paratyphi B], ParatyphiC [Salmonella paratyphoid caused by paratyphi C], Nosubstrainspecified [No specification of the organism that caused this Salmonella paratyphoid (Yes = Not specified and No = specified)], TPHA [Treponema pallidum Hemagglutination Assay as co-existing micro-organism or not (Yes = Positive and No = Negative)], Toxo [Toxoplasmosis gondii as co-existing micro-organism or not (Yes = Positive and No = Negative)], VDRL [Veneral disease research laboratory test status (Yes = positive and No = Negative)], HCV [Hepatitis C virus as co-existing micro-organism or not (Yes = Positive and No = Negative)], RF = Rheumatoid factor as co-test done (Yes = Positive and No = Negative)], HBV [Hepatits B virus as co-existing micro-organism (Yes = Positive and No = Negative)], CRP = C-reactive protein as co-test done (Yes = Positive and No = Negative)], ASLO = Anti-streptolysin O as co-test done (Yes = Positive and No = Negative)], Others = Other co-tests done (Yes = Positive and No = Negative)], XNone = No co-test done (Yes = done and No = not done)].
(XLSX)

S2 Data Amoebiasis data June 2018-Dec 2021. UniqueKey [Serial number], Healthfacilitylaboratory [Health facilities/laboratories (MMH = Mount Mary Hospital, RHB = Regional Hospital Buea, ACHC = Access Care Health Centre, MHC = Medicalized Health Centre, BTIHC = Buea Town Integrated Health Centre)], Age [Age in years], Sex [0 for male and 1 for female], Q4Year [Year test was carried out], Q5Month [Month test was carried out (0 = January, 1 = February, 2 = March, 3 = April, 4 = May, 5 = June, 6 = July, 7 = August, 8 = September, 9 = October, 10 = November, 11 = December)], Weekofthemonth [Week of the month test was carried out (0 = week 1, 1 = week 2, 2 = week 3, 3 = week 4, 4 = week 5, 5 = week 6)], Dayofthemonth = Date of the month test was carried out], PresenceofEntamoebahistolytica [Presence of Entamoeba histolytica or not (Yes = Present and No = Absent)], Colourofstool [Colour of stool (0 = Yellowish, 1 = Reddish, 2 = Blackish, 3 = Greenish, 4 = Brownish, 5 = Other colours)], Consistencyofstool [The consistency of the stool (0 = Soft, 1 = Watery, 2 = Formed, 3 = Semi-formed, 4 = Mushy, 5 = Mucoid and 6 = Others)], PresenceofWBC [Presence of white blood cells (Yes = Present, No = Absent and Missing = not specified)], PresenceofRBC [Presence of red blood cells (Yes = Present, No = Absent and Missing = not specified)].
(XLSX)

## Abbreviations

ACHC: Access Care Health Centre
BHD: Buea Health District
BTIHC: Buea Town Integrated Health Centre
COVID-19: Corona Virus Disease 2019
MMH: Mount Mary Hospital
MMHC: Muea Medicalized Health Centre
RHB: Regional Hospital Buea
